# Ultra-Fast High-Precision Metallic Nanoparticle Synthesis using Laser-Accelerated Protons

**DOI:** 10.1038/s41598-020-65282-9

**Published:** 2020-06-12

**Authors:** M. Barberio, S. Giusepponi, S. Vallières, M. Scisció, M. Celino, P. Antici

**Affiliations:** 1 Institut National de la Recherche Scientifique (INRS), EMT Research Center, 1650 boul. Lionel-Boulet, Varennes, Quebec, J3X 1S2 Canada; 2 ENEA, C. R. Casaccia, Via Anguillarese 301, 00123 Rome, Italy; 3 CELIA, Uni. of Bordeaux, 351 Cours de la Libération, Talence, 33400 France; 4 ENEA Fusion and Technologies for Nuclear Safety Department, C.R. Frascati - Via Enrico Fermi 45, Frascati, Italy

**Keywords:** Plasma-based accelerators, Ultrafast lasers

## Abstract

Laser-driven proton acceleration, as produced during the interaction of a high-intensity (I > 1 × 10^18^ W/cm^2^), short pulse (<1 ps) laser with a solid target, is a prosperous field of endeavor for manifold applications in different domains, including astrophysics, biomedicine and materials science. These emerging applications benefit from the unique features of the laser-accelerated particles such as short duration, intense flux and energy versatility, which allow obtaining unprecedented temperature and pressure conditions. In this paper, we show that laser-driven protons are perfectly suited for producing, in a single sub-ns laser pulse, metallic nanocrystals with tunable diameter ranging from tens to hundreds of nm and very high precision. Our method relies on the intense and very quick proton energy deposition, which induces in a bulk material an explosive boiling and produces nanocrystals that aggregate in a plasma plume composed by atoms detached from the proton-irradiated surface. The properties of the obtained particles depend on the deposited proton energy and on the duration of the thermodynamical process. Suitably controlling the irradiated dose allows fabricating nanocrystals of a specific size with low polydispersity that can easily be isolated in order to obtain a monodisperse nanocrystal solution. Molecular Dynamics simulations confirm our experimental results.

## Introduction

Laser-driven particle acceleration, in particular electron and proton acceleration, as obtained by intense laser irradiation, is a field that has attracted strong interest in the last few decades^1^. Several improved characteristics of these sources such as compactness, versatility and tunability, open a new potential for catalyzing diverse applications in different fields. Numerous high-power laser facilities that are upcoming, in construction or in commissioning, representing a cumulative impressive investment of almost 1 B€, have therefore the production of secondary sources as one of their key topics^2–8^. Concerning the field of laser-driven proton acceleration, as produced during the interaction of a high-intensity (I > 1 × 10^18^ W/cm^2^), short pulse (<1 ps) laser with a solid target^9^, several laboratories are working on setting up laser-driven proton sources for utilization of those novel particles^10–18^. Current applications of laser-accelerated particles (particularly protons)^19,20^ include their use as bright ultra-short neutron sources^21,22^, for producing warm-dense matter^23^, in medicine^24–27^, for picosecond metrology^28^, as diagnostics in the Cultural Heritage^29–32,88^, as well as for stressing and testing materials^33–36^.

Following a recent quest for new application using high-power in material science^37^, and profiting from the quick and intense heating generated by the laser-accelerated protons^38^, first attempts for their use in Advanced Material Synthesis have been made^39^. In this field, the control of nanoparticle and nanocrystal synthesis is currently a topic of fundamental importance. One of the foremost bottlenecks for many applications is the lack of a precise size in the production of nanoparticles^40^, and the possibility to produce solvent-free nanoparticles of any dimension^41^. The production details of the different nanoparticles strongly depend on the material, their size and their application^42–48^. As such, it is difficult to generalize state-of-the art technology and related results. Current commercially available monoatomic nanoparticles (e.g. gold)^49^ with diameter of 10–100 nm reach typically a size dispersion of about 30%^50^, (i.e. the size can be ±15% around the mean value), and, while cheap to manufacture, often come with the drawback of not being “pure”, since they are contained in solutions that produce a sometimes undesired and irremovable coating of the nanoparticles (NPs) - not always appropriate for the suggested applications. In addition, current technology is still lacking methods for producing some multi-atomic particles, or specific nanoparticles below a certain dimension (see later). Thus, there is market need for (quicker) manufacturing techniques able to produce a larger variety of nanoparticles that are more versatile in its utilization^51^: Several nanotechnology centers^52^ and government organizations^53^ are requesting proposals for the fast production of a large quantity of solvent-free isolated nanoparticles with higher precision (the required dispersion is ≤10%), where examples can be found in the production of ultra-small silica-organic hybrid nanoparticles “that have the potential to dramatically impact the way we diagnose and treat cancer patients based on their favorable physicochemical and imaging properties”^52^, or the production of 60 nm gold nanocrystals with very low dispersion for the detection of early tumors in child brains since improving different imaging techniques^54^. In both cases, higher production costs are justified since they allow overcoming important technological bottlenecks or might enable the production of yet non-existing nanoparticles (a similar example where high economic effort is justified, is the proton therapy, a very expensive cancer treatment, however unique for curing specific tumors (e.g. brain tumors)).

The main problem for producing low-dispersion nanocrystals is identifying the parameters to generate the conditions of temperature and pressure needed to produce well-defined structures in very short timescales (fs-ns). These timescales are essential for the nucleation of particles with dimensions in the 5–200 nm range^55–57^.

This can be obtained by irradiation of matter using an energetic proton beam with short duration, such as generated by laser-driven proton acceleration. In a preliminary proof-of-principle experiment^39^ we introduced a physical method for the synthesis of micro-crystals based on the ablation of material using an ultra-short ultra-intense laser-generated proton beam. The ablation process, which produces a plume for NP nucleation and aggregation, is the result of an explosive boiling in the bulk target. While in the work we were only able to use the method for the production of micro-crystals (in particular mono-atomic), which is of very little use for applications, the situation is completely different if the synthesis process could be adapted to produce crystals in the nanometer regime. In the method that we propose for this purpose, a solid material with a low melting temperature is irradiated by a laser-accelerated proton beam. This intense irradiation provokes ablation of the material in few tens of ns generating the better conditions for the nucleation of nanomaterials. This is different from typically known laser-based nanoparticle synthesis methods. In conventional ablation technologies (such as pulsed laser ablation (PLD) and laser ablation in solution (LASIS)), an UV or IR laser with a pulse duration in the order of ns and a frequency of tens of  Hz irradiates a bulk target for tens of seconds. The laser-matter interaction generates the ablation of atoms from the irradiated surface and the atoms. In the PLD, the NPs aggregate on a solid target surface, in LASIS they aggregate in a colloidal solution; in our technique the atoms with high kinetic energy aggregate in the plume, generating clusters of nanoparticles.

Contrary to conventional laser ablation technologies where photons are heating the material, when using laser-accelerated protons the sample is irradiated for a duration of only up to a few tens of ns. This duration is produced by the debunching of the ions that are instantaneously accelerated from the source with different energies and therefore arrive on the secondary target at different instants of times. This allows reaching very quickly thermodynamic conditions that are between the boiling point and the critical point. The high-energy proton beams are generated by the commonly known as Target-Normal Sheath Mechanism (TNSA)^58^, occurring when a high-intensity (I > 10^18^ W/cm^2^), short-pulse (duration <1 ps) laser, commercially available these days, hits a target (*proton source target*) with micrometric thickness under vacuum condition. Despite its limits^59^, it is currently the most routinely available acceleration mechanism. Our setup allows generating in the proton-irradiated material bulk (*working or plume target/sample*) temperature and pressure conditions that are not available in conventional nanomaterial synthesis laboratories even using industrially produced ion beams, due to their short duration^60^. The thermodynamic conditions produced by the laser-accelerated protons trigger the nucleation process, and in particular the nucleation of small dimension crystals with a high control in crystallinity and dimensions. The interaction between the laser-generated proton beam and the low-boiling material detaches atoms and ions from the target surface and emits them in a plume, producing the formation of nanoparticles with very high mean kinetic energy. The detached particles are then deposited on nearby cold solid surfaces for obtaining nano- and microstructuration.

The Laser-Accelerated Proton-Driven Ablation (LAPDA) growth process occurs in three steps, as described in ref. ^39^. The proton-induced heating occurs very locally and the solid working targets dissipate heat very efficiently. As a result, the detachment of the material by the thermal mechanism is occurring only in a region almost corresponding to the irradiating proton spot (in the order of mm^2^). The transverse (i.e. orthogonal to the surface normal) heat dissipation is relatively unimportant because the proton bunch is very short. The ablation mechanism provoked by the proton heating is strongly influenced by the energy deposited by the proton beam into the working target bulk and on the duration of interaction. Varying the distance between the proton source and the material sample can control the heating of the surface: the intrinsically ballistic spray of the divergent protons produces a lower proton flux at larger distances and outer regions from the irradiation center. In addition to this, the proton beam debunches (i.e. becomes longer in time) with increasing distance since the different particle energies tend to widen the bunch length when travelling over longer distances.

In this paper we demonstrate that Laser-Accelerated Proton-Driven Ablation allows producing nanocrystals of different materials with tunable size, ranging from a few nm to hundreds of nm, very low dispersion and in a single sub ns laser pulse . We show that the nanocrystal size is determined by the local heating effect produced by the laser-generated protons and allows for an a-priori determination of the particle size. The nanoparticle dispersion depends on the heating uniformity; therefore a uniform heating process produces nanocrystals of the same size. The generated nanocrystals can easily be detached from the surface, e.g. by performing a sonication in a polar solvent bath for a few minutes. The detached particles can then be mixed into a colloidal solution.

Simulations using an Energy Deposition model and Molecular Dynamics Code confirm our experimental findings and the underlying modeling.

## Results and Discussion

**Figure 1:**
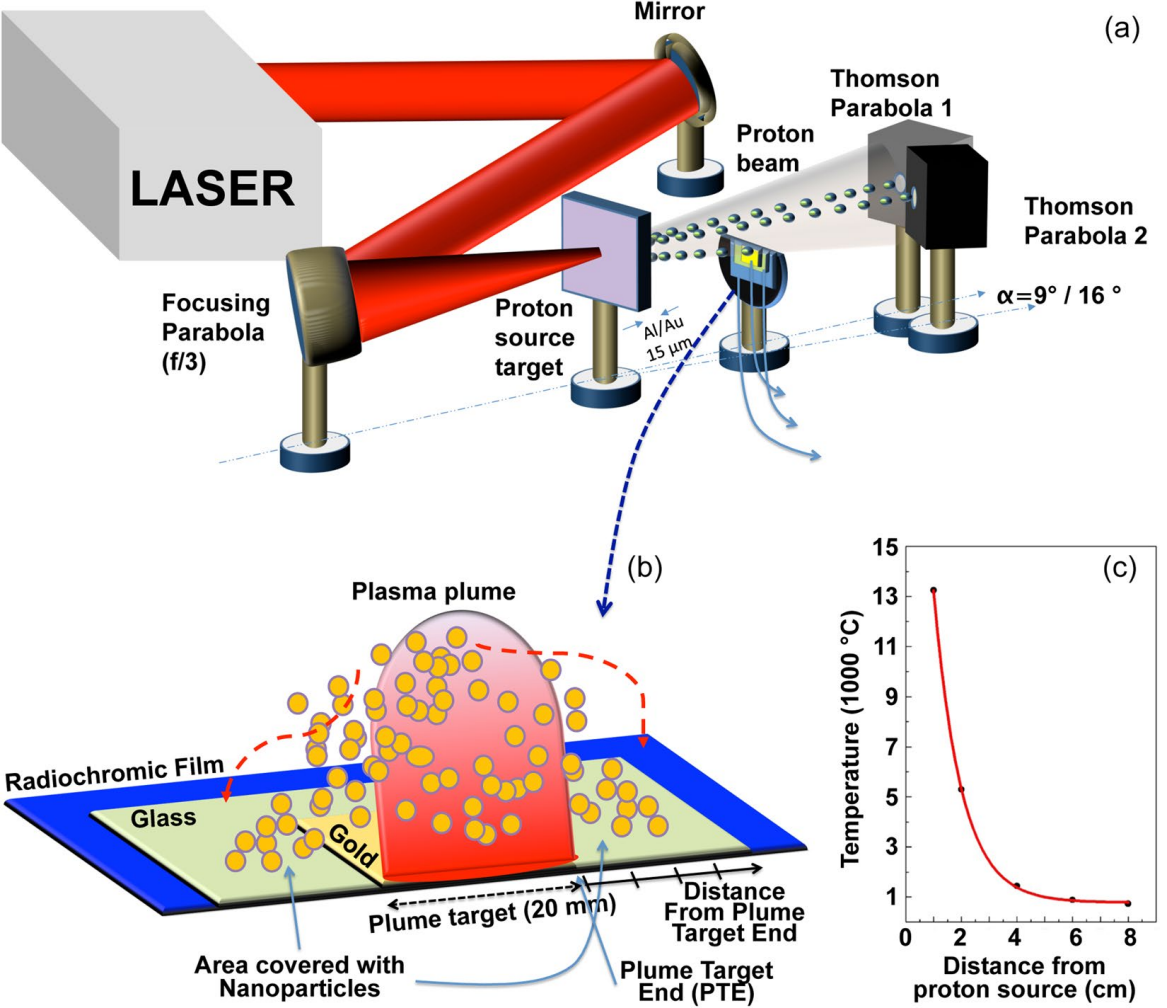
(**a**) Sketch of the experimental setup and (**b**) sketch of the plume expansion on the target. (**c**) Temperature as function of source/target distance for the TITAN laser, as resulting from the Energy Deposition Code. The Plume Target End (PTE) is located at the end of the working (plume) target that is 20 mm in length.

The experiments were performed on the TITAN laser of the Jupiter Laser facility (operating in the Lawrence Livermore National Laboratory (LLNL) located in California) (see Fig. 1a and experimental setup details in the Experimental methods) and at the ELFIE laser operating in the LULI facility located in Palaiseau (France). The TITAN laser produces pulses of about 220 J in 700 fs. This laser operates at a wavelength of 1.054 µm^61^ while the ELFIE laser delivers pulses of about 20 J in 350 fs at a wavelength of 1.057 µm. In both setups, a f/3 parabola was focusing down the laser to a beam waist of about 9–10 µm Full-Width-Half-Max (FWHM). The laser was interacting with a solid aluminum target (*proton source target*) of thickness 15 µm, accelerating protons at the back of the target surface. Typical laser-generated proton spectra obtained during the experiment are shown in the Experimental methods. The laser-accelerated protons were impinging onto a commercially available solid gold sample (purity 99.99%) with dimensions of 10 × 20 mm and of thickness 250 µm located at a distance ranging from 0.5 to 8 cm from the proton source (see Experimental methods). The gold sample (*working or plume target*) to be irradiated by the laser-accelerated protons was fixed, horizontally in the center and vertically close to the edge) on a microscope glass of dimensions 7.5 × 2.5 cm in order to catch all the nanoparticles generated by the gold sample in the plasma plume on the glass surface (*collector*), while also enabling detection of part of the proton beam (see Fig. 1a,b).

Using an Energy Deposition model^34^ in which we coded the experimentally measured proton source parameters, we could evaluate the temperature near the surface in order to find the optimum conditions for our LDPA. From simulations (see Fig. 1c) we obtained that for the TITAN laser the surface temperature stays above the gold boiling point (around 2800 °C^62^) for distances shorter than around 3 cm, between the boiling and the melting point (around 1000 °C^62^) for distances between 3 and 5 cm, and then decreases to temperatures of only a few hundreds of degrees at distances around 7–8 cm in the central axis of the proton beam. For the ELFIE laser these distances were respectively 2, 4 and 6 cm. From these results, we could deduce that the temperature range for the explosive boiling is related to an optimized proton-source-to-sample distance occurring between 2 and 4 cm. For shorter distances, the higher temperature completely destroys the target surface, causing the ablation of macro fragments from the target bulk and preventing the particle synthesis. For longer distances, up to a distance of 6–7 cm, the bulk material undergoes a “lighter” boiling process, yielding to a smaller plume and a much lower number of generated nanocrystals: for distances above 6–7 cm the temperature is not sufficiently high for generating any boiling. We note that most of the proton energy is deposited within the first tens of microns of the material depth (according to  the SRIM data base, protons with a mean energy of 2 MeV reach a depth of up to 15 µm) and then decreases very rapidly with increasing depth in the sample. This is due to a high number of low energy protons in the spectrum (<2 MeV), protons that have a very high stopping power in this energy range for the used working target material (in our case gold) and deposit it very locally (Bragg peak). Moreover, we note that due to the cylindrical symmetry and almost Gaussian profile of the proton beam the temperature condition for the explosive boiling is reached at radial distances of up to a few mm from the proton beam center. The exact size depends on the distance from the proton source, but is around 5 mm for a proton source to gold working target distance of 2 cm.

**Figure 2:**
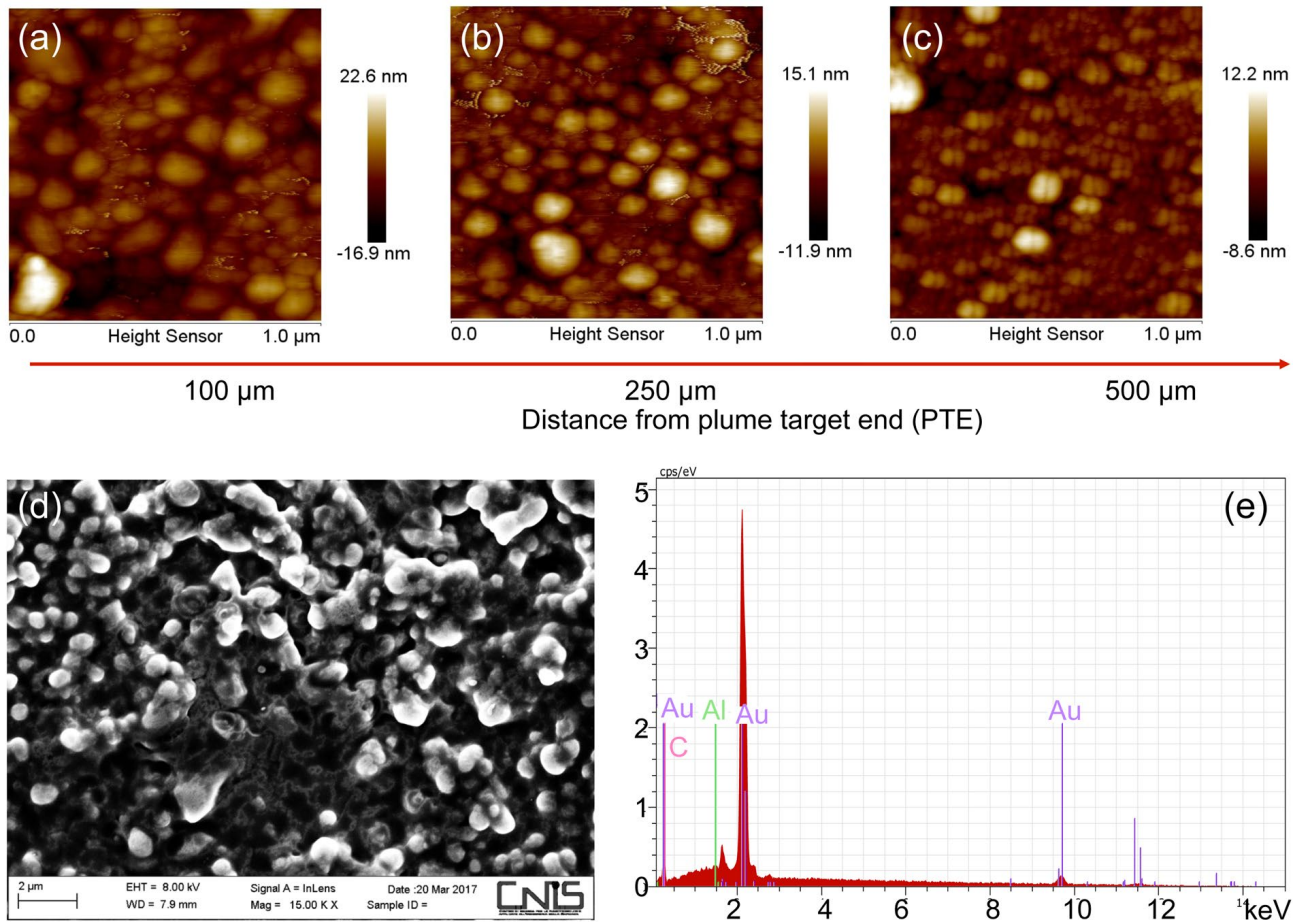
(**a–c**) AFM images taken at different transversal distances from the Plume Target End (PTE) (for the sample located at a distance of 5 cm from proton source); (**d**) SEM image taken at a distance of 2 cm from the proton source and a transverse distance of 250 µm from the PTE; (**e**) EDX analysis conducted under SEM conditions for particle synthesized in the same conditions (2 cm from proton source and transverse distance of 250 µm from the PTE).

The predictions by the Energy Deposition Code were confirmed by the experiments. In the experiments we used the above-mentioned optimum distance condition between the proton source and the irradiated gold sample. Considering in the following the shots performed on the TITAN laser, Atomic Force Microscope *(*AFM) images (Fig. 2a–c) and a SEM image (Fig. 2d) taken from the collector glass surface located close to the ablated gold working target show the formation of a nanostructure composed by particles with dimensions ranging from hundreds of nm down to few nm for distances between the source and the collector glass between 2 and 7 cm. Energy Dispersive X-ray Analysis (EDX) measurements taken on the nanostructures located on the collector glass surface (Fig. 2e) show a bulk composition which consists of a thick layer of gold nanoparticles deposited onto the collector glass surface.

**Figure 3:**
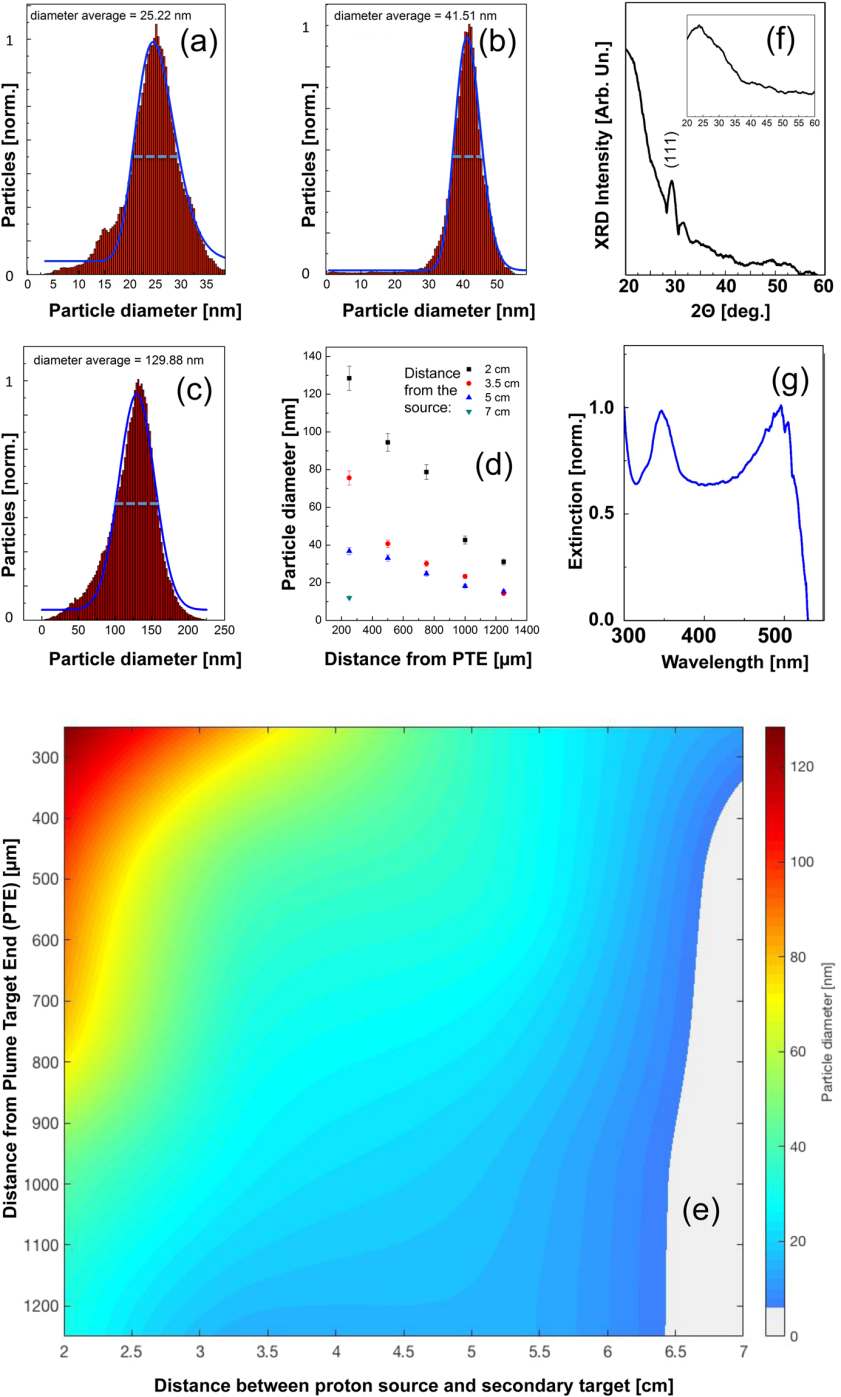
(**a**) Histogram of the particle dimensions for particles obtained at a distance of 5 cm from the proton source, a transverse distance of 750 μm from the PTE. (**b**) Histogram of the particle dimensions for particles obtained at a distance of 2 cm from the proton source, a transverse distance of 1000 μm from the PTE. (**c**) Histogram of the particle dimensions for particles obtained at a distance of 2 cm from the proton source, 250 μm from the plume end. (**d**) Particle dimensions vs. transverse distance from the PTE, as function of distance from the proton source. (**e**) 3D particle diameter map as depending from the distance from the proton source and from the distance of the plasma plume. The map is interpolated from experimental data, some of those displayed in panel (**d**). (**f**) XRD and (**g**) Plasmonic analysis for particle synthesized at 2 cm from proton source (as example for all). The inset in the panel (**f**) shows the XRD spectrum of the pristine target. All results are related to irradiations performed on the TITAN laser.

A detailed analysis of different zones within the surface onto which the nanoparticles were deposited is given in the Fig. 3. The particle diameter was evaluated by analysis of about 1000 NPs produced with the same shot/proton conditions (see details in the Experimental methods). The indicated values are mean value and their  standard deviation, computed by the OriginLab software („simple statistic toolkit“) using values obtained by the AFM software. The mean values and variances of the nanoparticle diameters (as shown in the histograms) were evaluated by a statistical analysis on the different AFM images taken at different positions within the glass (or copper) substrate. Figure 3a–c shows histograms of the particle diameter analysis for different distances of the working target from the proton source, and for different transverse distances from the plume  target end (PTE). The PTE is the position until where the plasma plume is generated during the interaction . Typically it corresponds  to the end of the working target - see its definition in Fig. 1b. The data shown in Fig. 3 are taken respectively for panel (a) at a source distance of 5 cm and transverse distance from the PTE of 750 µm, for panel (b) at a source distance of 2 cm and transverse distance from the PTE of 1000 µm and for panel (c) at a source distance of 2 cm and a transverse distance from the PTE of 250 µm. One can see a narrow dispersion of the particle diameters, which, as obtained over a statistical analysis (see Experimental methods), reaches a standard deviation of about 10%. A more systematic study of the dependence of the distance of the produced nanoparticles from the plume target end and the dependence of the produced nanoparticles from the distance of the proton source is indicated in Fig. 3d. In Fig. 3d we show the particle dimension when varying the radial dimension but fixing the distance from the proton source to different distances, namely 2, 3.5, 5, 7 cm. One can see that the particle diameter decreases with increasing distance from the plume target end. In the case of a source distance of 2 cm the size ranges  from about 130 nm for a distance from the plume target end of 250 µm down to about 25 nm for a distance from the plume target end of 1250 µm. We find an almost linear tendency line that allows for tunable nanoparticle sizes when selecting the nanoparticles from zones at different distances from the plume target end. However, there is also a scaling law associated to the distance of the produced nanoparticles from the proton source. Figure 3d shows also that the largest particles with a diameter of 130 nm are obtained at closer distances from the proton source (about 2 cm), while smaller particle sizes around 5 nm are obtained for distances of about 7 cm. In between this distance range, we see a scaling, and in particular an increasing particle dimension with decreasing distance following an exponential law. Linking the information about the radial distance and proton source distance dependences regarding nanocrystal size allows producing a 3D tunable nanoparticle-dimension map, in which the desired nanoparticle diameter can, at known proton irradiation pattern, be predetermined (see Fig. 3e). This enables  the quick production of a-priori-established high-precision tunable nanoparticles within the range 5–130 nm. The size and monodispersity of the produced nanocrystals depend on the heating effect produced by the deposited proton dose, which - due to the characteristics of the TNSA acceleration scheme^63^ - is of cylindrical/toroid geometry around the proton beam center. As such, in the present case, the monodispersity of the nanocrystals is determined by the size of the toroid structure around the beam center. A more efficient way is to shape the proton beam in order to make it more uniform. Uniformity of the beam is currently a very active field of investigation for laser-plasma acceleration, given in particular the outlook for medical application of these laser-driven proton beams (e.g. proton therapy^24^) where uniform doses are required (that can be achieved by either acting on the single beam or by adaptive beam scanning). Several group have developed tools for providing users with requested beam characteristics and uniform doses^64–67^. The absence of particles for distances lower than 2 cm from the proton source, as anticipated by the Energy Deposition Code, is supported by the analysis of the proton-irradiated working gold target producing the plasma plume, which presents a strong ablation and peripheral melting by the proton irradiation, with many craters having dimensions ranging from hundreds of nm to few μm^34^. The strong ablation of the surface confirms the reaching of temperatures indicated by the Energy Deposition Code simulations. Finally, the absence of particles for distances greater than 7 cm and the fact that the gold surface stays unmodified after the proton irradiation, confirm that for these high distances the temperatures in most part of the irradiated gold target are much lower than the melting point and cannot induce the thermodynamic conditions for the particle synthesis. Large variations in nanoparticle dimensions (from hundreds down to few nanometers) indicate that the atom aggregation in the plume is strongly related to the energy deposited in the bulk and in the consequently different detachment mechanism from the bulk. The decrease of the deposited dose with increasing source-to-target distances generates a slower detachment mechanism of the atoms from the surface. Moreover, the lower deposited dose induces the ablation of particles with lower kinetic energy. Therefore, higher deposited dose produces a very dense plume with highly energetic atoms, which favors the aggregation of a high number of atoms and then the synthesis of large particles. Inversely, lower deposited dose indicates low-density plume with low atom mobility, which causes a decrease in the aggregation and in the final particle dimensions. The changes in the dimensions of the nanoparticle with distance from the plume target end (i.e. the distance where the proton irradiation has no further effect) can be related to the kinetic energy of the synthesized particles. Bigger particles with lower velocity reach the glass surface very close to the plume end, while small particles with higher velocity are deposited on more distant distances.

Particles with different dimensions can be isolated and transferred in a colloidal solution, making them usable for applications (more details about how to do this are indicated in ref. ^39^). To verify this in the current experiment, we placed copper plates (dimensions of 2 × 10 mm) at different distances from the plume end and made sure we were collecting only particles with similar size. After the shot the copper plates, completely covered by NPs, were inserted in an acetone bath and sonicated for 3 hours for detaching the particles from the surface. The obtained colloidal solution was analyzed by AFM microscopy. 2D and 3D AFM images of NPs produced in colloidal solutions are shown in the Experimental methods (Fig. 12).

From the analysis of AFM and SEM images we can calculate for one laser pulse a production in terms of particle density (for nano-particles with a diameter of 20 nm) of 150 NPs/μm^2^. Considering that we can cover with regular particles a surface in the order of cm^2^ (the final surface depends on the intensity of the available laser and its capability to produce an intense proton beam, but the beam being divergent at 25° half angle, at a distance of 4 cm, the beam diameter is about 3.5 cm, i.e. produces a surface of 10 cm^2^) we can produce for each laser pulse about 1.5 × 10^11^ NPs with the same diameter (the number of particles changes very little between the different measured diameter sizes). High-power lasers can be of different repetition rates. Commercially available high-power lasers generating high-energy protons can now routinely reach 10 Hz^37^. Hence, considering the use of a 10 Hz laser (10 pulses/s), the total production rate would become 1.5 × 10^12^ NPs/s, provided there is 10 Hz availably for both proton source targetry and plume target.

Further analysis of the nanoparticles using X-ray diffraction (XRD) data (see Fig. 3f) shows the presence of gold crystals with orientation (111). The crystal structure is absent in the XRD spectrum of the pristine target (see the inset in Fig. 3f) indicating that the energy conditions for the plume support the crystalline aggregation of atoms. Finally, the plasmonic resonances measured on about 10^8^ gold particles deposited on one sample of glass substrate confirm the simultaneous presence of particles with different dimensions. The plasmonic spectra were evaluated following the exact Mie Theory in Refs. ^68,69^, which allows evaluating the nanoparticle diameter. It is well known, that the surface plasmonic resonance (SPR) emission of NPs is strictly related to the particle dimensions. In details, the theoretical SPR spectra of a gold NP collection typically show a series of bands ranging between 350 nm and 650 nm, and each band corresponds to the presence in the collection of particles with specific dimensions^70^. The plasmonic spectrum in Fig. 3g shows two bands located at 350 nm and at 490 nm, which corresponds to particles with dimensions of a few nm (resonance at 350 nm) and about 100 nm (resonance at 490 nm) (see Experimental methods)^38,39,71^.

**Figure 4:**
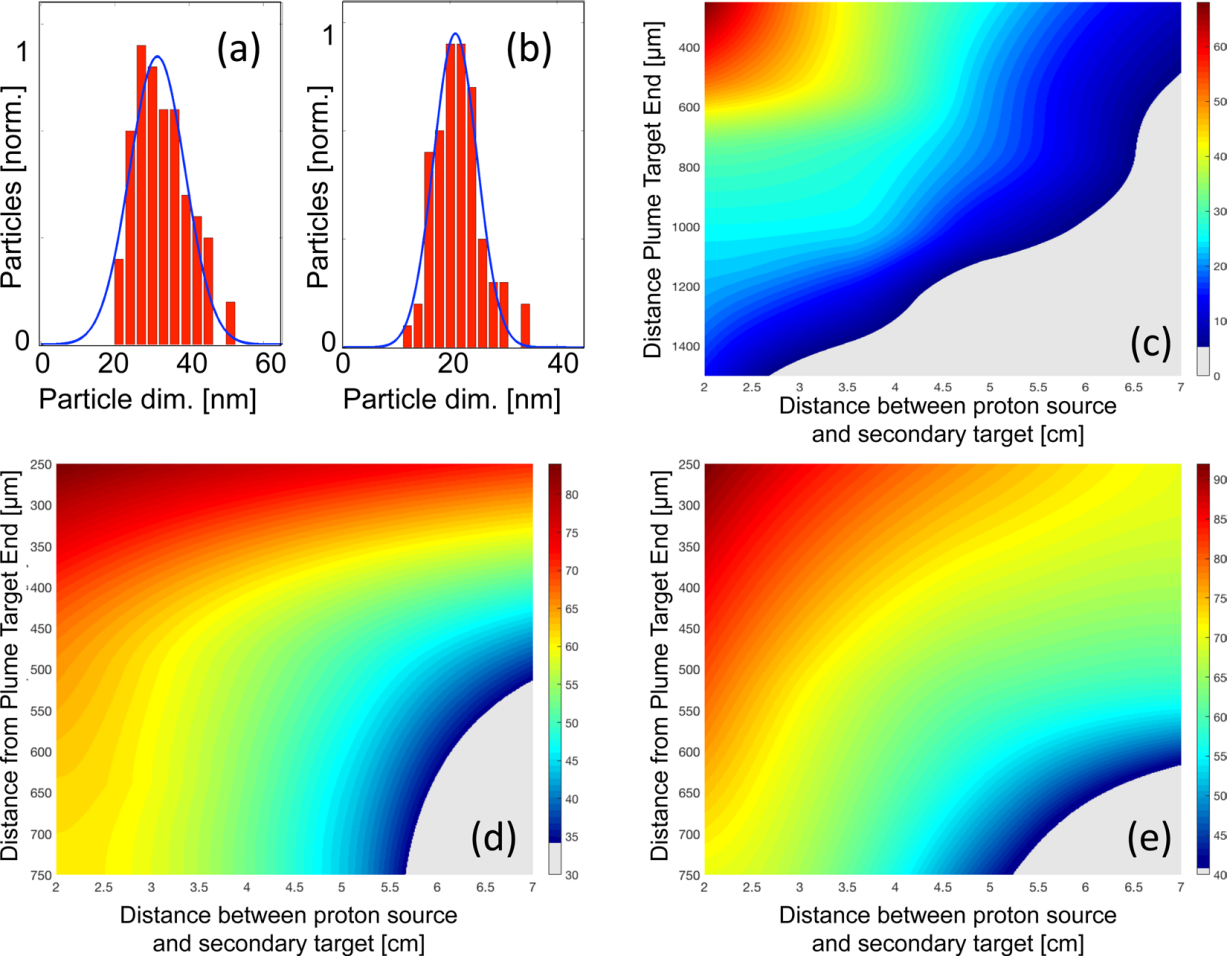
(**a**) Histogram of the particle dimensions for particles obtained at a distance of 2 cm from the proton source, a transverse distance of 750 µm from the PTE (histograms are normalized to maximum particle number). (**b**) Histogram of the particle dimensions for particles obtained at a distance of 5 cm from the proton source, a transverse distance of 250 µm from the PTE. (**c**) Particle dimensions (in nm) vs. transverse distance from the PTE, as function of distance from the proton source for a gold target. (**d**) particle dimensions vs. transverse distance from the PTE, as function of distance from the proton source for a copper target. (**e**) particle dimensions vs. transverse distance from the PTE, as function of distance from the proton source for a aluminum target. All results are related to irradiations performed on the ELFIE laser.

Our findings are confirmed by the results obtained on the ELFIE laser (see Fig. 4a-e) using the same methodology adopted before . On this laser the generated gold particles have dimensions ranging between 68 nm (for an irradiation at a distance of 2 cm from the source) and 10 nm (for a distance of 7 cm from the source) in diameter. For a distance of 2 cm from the proton source, nanoparticles up to a transversal distance of 1500 µm from the plume end were deposited, with a diameter of 15 nm (see Fig. 4c). As identified by the statistical analysis (see examples on Fig. 4a,b) the dispersion of these particles is still with similar standard deviation as on the TITAN laser.

The different nanoparticle ranges for this laser compared to those obtained on the TITAN laser are justified by the fact that the proton spectrum obtained on the ELFIE laser is different from that obtained on the TITAN laser.  The ELFIE has fewer particles, and therefore the heating process on the material sample is different.

We also tested the applicability of our method for other materials such as aluminum and copper using again the same experimental setup and methodology. The nanoparticle sizes range respectively from about 35 to 90 nm for both, aluminum and copper (Fig. 4d,e). EDX analysis under SEM conditions confirms that the layer is almost entirely made of aluminum and copper nanostructures (see Experimental methods). The difference of the obtained nanoparticle range is again to be found if a different heating process, due to different stopping power of these materials. Simulations using the energy deposition code show a reduced temperature increase for aluminum and copper when irradiated by the proton beam.However, this effect is compensated by the lower boiling temperature of aluminum and copper, which still enables triggering the process even at a lower temperature. Moreover, the aluminum and copper atoms are lighter compared to gold, leading to a higher velocity of the ablated particles present in the expanding plume. Hence, for the case of aluminum or copper materials, the proton irradiation induces a higher mobility of the atoms in the plume, leading to the generation of larger nanoparticles that aggregate on the surrounding glass surface.

**Figure 5:**
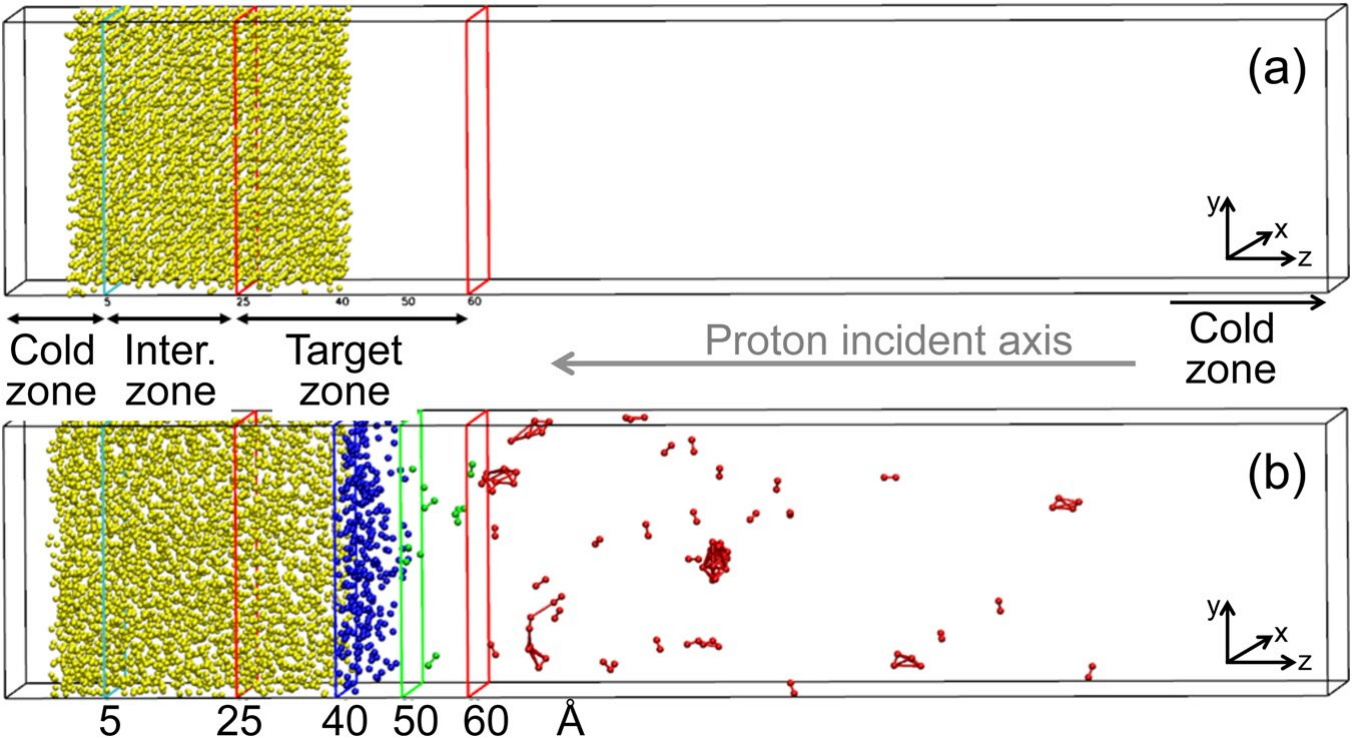
(**a**) Snapshot of the gold system used as starting configuration for the simulations. Yellow spheres represent gold atoms. The cold zone is on the left side of the vertical cyan line (*z* ≤ 5 Å). The intermediate zone is within 5 Å < *z* ≤ 25 Å. The target zone, in which the gold (100) surface will undergo the explosive boiling, is between the red lines (25 Å < *z* ≤ 60 Å). (**b**) Snapshot of the system at t_2_ = 80 ps of the simulation in which the target zone is at T^*^ = 3500 °C. Blue atoms (40 Å < *z* ≤ 50 Å) represent the melted surface; green atoms have 50 Å < *z* ≤ 60 Å; red atoms (*z* > 60 Å) represent the plume.

To confirm the hypothesis and correctly understand the nanoclusters formation mechanism via evaporation of atoms and their aggregation in the plasma plume, we performed extensive classical Molecular Dynamics (MD) simulations to analyze the behavior of a gold surface in which a large amount of energy is deposited in a very short time frame. In the simulation, a gold (100) surface (see Experimental methods) has been heated during 800 ps at a fixed temperature *T* = 500 °C (Fig. 5a) in order to have a high temperature starting configuration in the crystalline phase. Three regions of the simulation cell were chosen with different temperatures: the *cold* zone (*z* ≤ 5 Å) in which the temperature is kept fixed at *T* = 500 °C to impose a bulk like behavior of the atoms; the *intermediate* zone (5 Å < *z* ≤ 25 Å) in which there is no temperature constraint; the *target* zone (25 Å < *z* ≤ 60 Å) in which a controlled amount of energy is supplied in tens of ps, as occurs during the experiment. We indicate with *t*_1_ the simulation time to reach a prefixed temperature T^*^ in the target zone: *t*_1_ can vary from 4 ps to 60 ps, depending on the final T^*^ to be reached. T^*^ temperatures range from 800 °C to 3500 °C exploring the surface behavior in the melting regime and beyond.

For each simulation, once the target temperature T^*^ is reached, a further MD simulation is performed for 20 ps (from *t*_1_ to *t*_2_) keeping the temperature T^*^ constant in the target zone. This time interval is sufficiently long to observe the detachment of atoms (early plume formation) and the cluster formation in the plume above T^*^ = 2500 °C.

**Figure 6:**
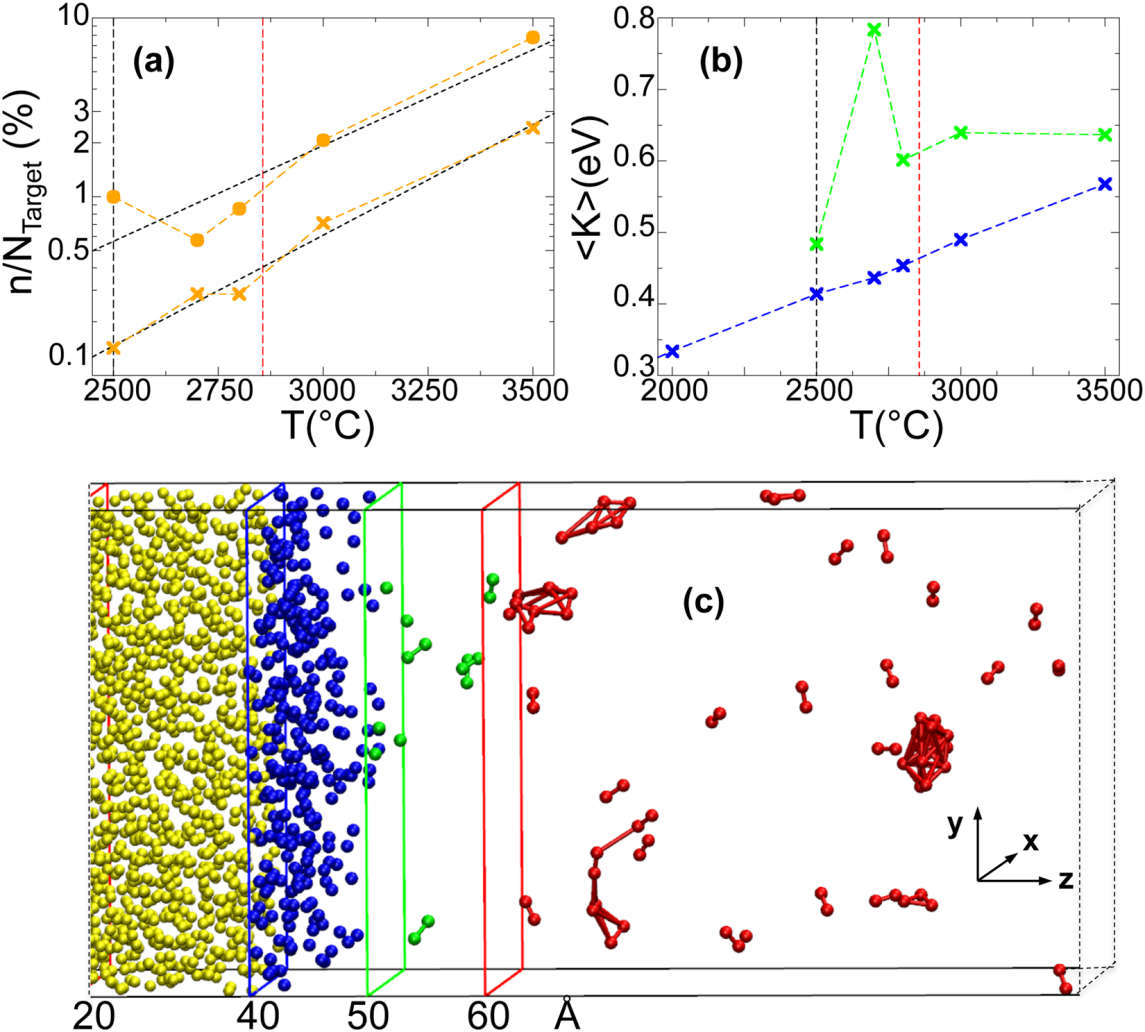
(**a**) Fraction (*n/N*_target_) of detached atoms from the surface (with *z* ≥ 50 Å) at the time *t*_1_ (orange crosses) and at the time *t*_2_ (orange dots) vs. temperature (T) of the target zone. Dashed black and red lines refer to the lower T^*^ for the detachment of gold atoms and boiling temperature of gold, respectively. (**b**) Mean values of the kinetic energy <*K* > of atoms in the two zones of the simulation cell (as described in Fig. 5b) vs. temperature (*T*) of the target zone. Dashed vertical lines as above. (**c**) Zoom of Fig. 5b to highlight the detachment of gold atoms and the formation of clusters.

The atoms detachment occurs in form of dimers and this process becomes more evident by increasing the temperature. In the higher temperature limit of 3500 °C the entire process is fast enough to allow for the formation of small clusters outside the target zone, which can be seen on the right side of the simulation cell: Fig. 5b shows the numerical system at the time *t*_2_ and T* = 3500 °C. To characterize the cluster formation process, we report in Fig. 6a the fraction *n/N*_target_ versus T* at the simulation time *t*_1_ and *t*_2_, where *n* is the number of detached atoms (with *z* ≥ 50 Å) and *N*_target_ = 1400 is the number of gold atoms inside the target zone at the beginning of the simulation: orange crosses refer to the time *t*_1_, whereas orange dots  refer to the time *t*_2_. It is interesting to note that both graphs in Fig. 6a exhibit a linear trend on a logarithmic scale indicating a significant effect of the supplied energy in short time frames on the temperature increase. Indeed, at the end of the simulation with T* = 3500 °C there are about 10% of *N*_target_ atoms segregated from the surface and involved in the cluster formations. These results can be verified by visual inspection of the system by zooming in the target zone (Fig. 6c).

To further characterize the results, we identify the atoms located inside two areas of the target zone. The two groups of atoms are dynamically selected based on their *z* coordinate (see Fig. 5b): atoms on the surface 40 Å < *z* ≤ 50 Å (blue atoms), and atoms that leave the surface 50 Å ≤ *z* < 60 Å (green atoms). For each simulation, we evaluated the distributions of the kinetic energy of these two groups of atoms averaged in the time interval *t*_1_ – *t*_2_ (i.e. during the detachment phase). Figure 6b reports the mean values of the kinetic energy <*K* > in function of the temperature of the target zone. With regard to the first group of atoms, a linear growth of the mean kinetic energy is observed (blue symbols). On the contrary, for the other group of atoms (green symbols), it is difficult to attribute a single tendency line to the mean kinetic energy . The trend is likely to occur due to superposition of two different phenomena, i.e. the interplay between an increasing supplied energy for higher temperatures, and an intensifying energy transformation occurring during the clustering phase. This clustering phase occurs at higher temperatures and reduces the kinetic energy of the atoms. In Fig. 6c the red marked atoms with *z* ≥ 60 Å depict their tendency to aggregate in clusters confirming the experimental findings of cluster aggregation.

**Figure 7:**
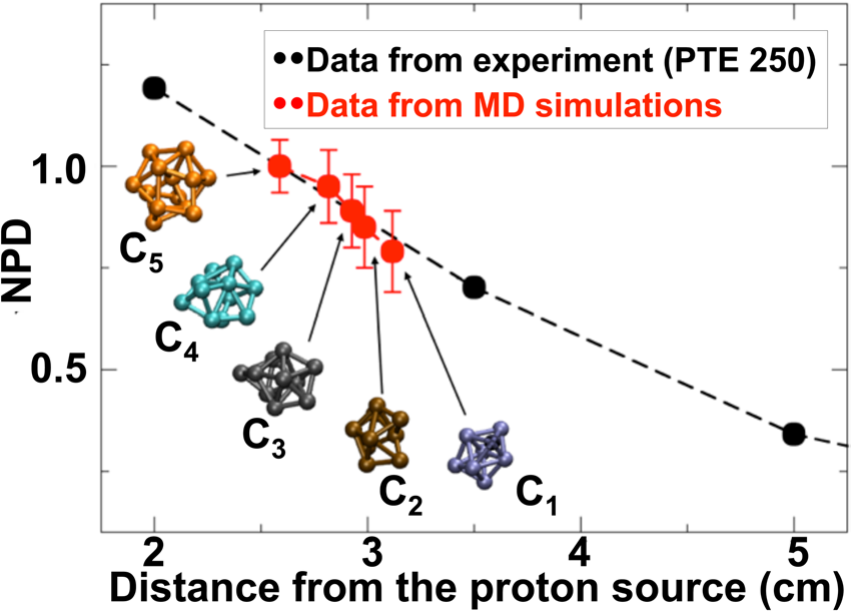
Normalized Particle Diameter (NPD) vs. distance from proton source. Black dots  refer to experimental data obtained at 250 μm from the Plume Target End (PTE). Red dots  refer to MD simulation data in the range 2500 °C < T^*^ < 3500 °C (distance from the proton source in the range of 3.1–2.6 cm). C_1_, C_2_, C_3_, C_4_ and C_5_, are snapshots of the clustersextracted from the simulations.

Finally, to have more quantitative indications about the effect of the temperature on the cluster size, the MD simulations were further extended for 300 ps in the range 2500 °C < T* < 3500 °C. This time interval is long enough to observe the formation of clusters at all temperatures already before the atoms reach the cold zone located in the far right end of the simulation box. For each temperature, the nanoparticle diameter was estimated and time-averaged on the available clusters in each simulation (for details see the Experimental methods: an example of simulations sequence is displayed in Fig. 10, the different clusters obtained at the end of the simulations are reported in Fig. 11, or in the related video materials indicated in the Experimental methods). By using the relationship between temperature and distance displayed in Fig. 1c, it is possible to find the experimental nanoparticle diameters (for the different distances from the proton source) versus temperature whose slope can be compared with the numerical findings. However, since the sizes of both simulated and experimental systems are on different scale lengths, particle diameters are normalized before being superimposed in the same figure (Fig. 7). MD simulations data (red dots ) are normalized with respect to the diameter of the cluster *C*_*5*_ corresponding to T* = 3500 °C (2.6 cm from the proton source); experimental data (black dots ) relative to the distance from PTE = 250 μm (see Fig. 3d,e) are normalized with respect to the (interpolated) value corresponding to the experimental data obtained 2.6 cm from the proton source. We observe a very good agreement between the two curves in Fig. 7, which confirms that our numerical model catches the intrinsic behaviour of the experiments.

## Conclusions

In this paper, we present a technique for a quick a-priori production of nanoparticles using the recently introduced laser-driven proton-ablation mechanism. We present experimental results validating that we are able to produce isolatable metallic nanoparticles in the range of 5–130 nm with very high precision and in a single sub ns laser pulse, nanoparticles  that can be put in a colloidal solution. Our results are confirmed by theoretical modeling, which relates the properties of the produced particles with the energy deposited by the proton beam on the bulk surface used for the ablation. In details, using Energy Deposition Codes and Molecular Dynamic simulations we demonstrate that changing the energy deposited on the surface (e.g. by varying the setup geometry) allows synthesizing nanoparticles with different dimensions and that the number and the energy of the particles evaporated from the surface are strictly dependent on the proton-deposited dose.

## Experimental methods

### Laser experiments and diagnostics

**Figure 8:**
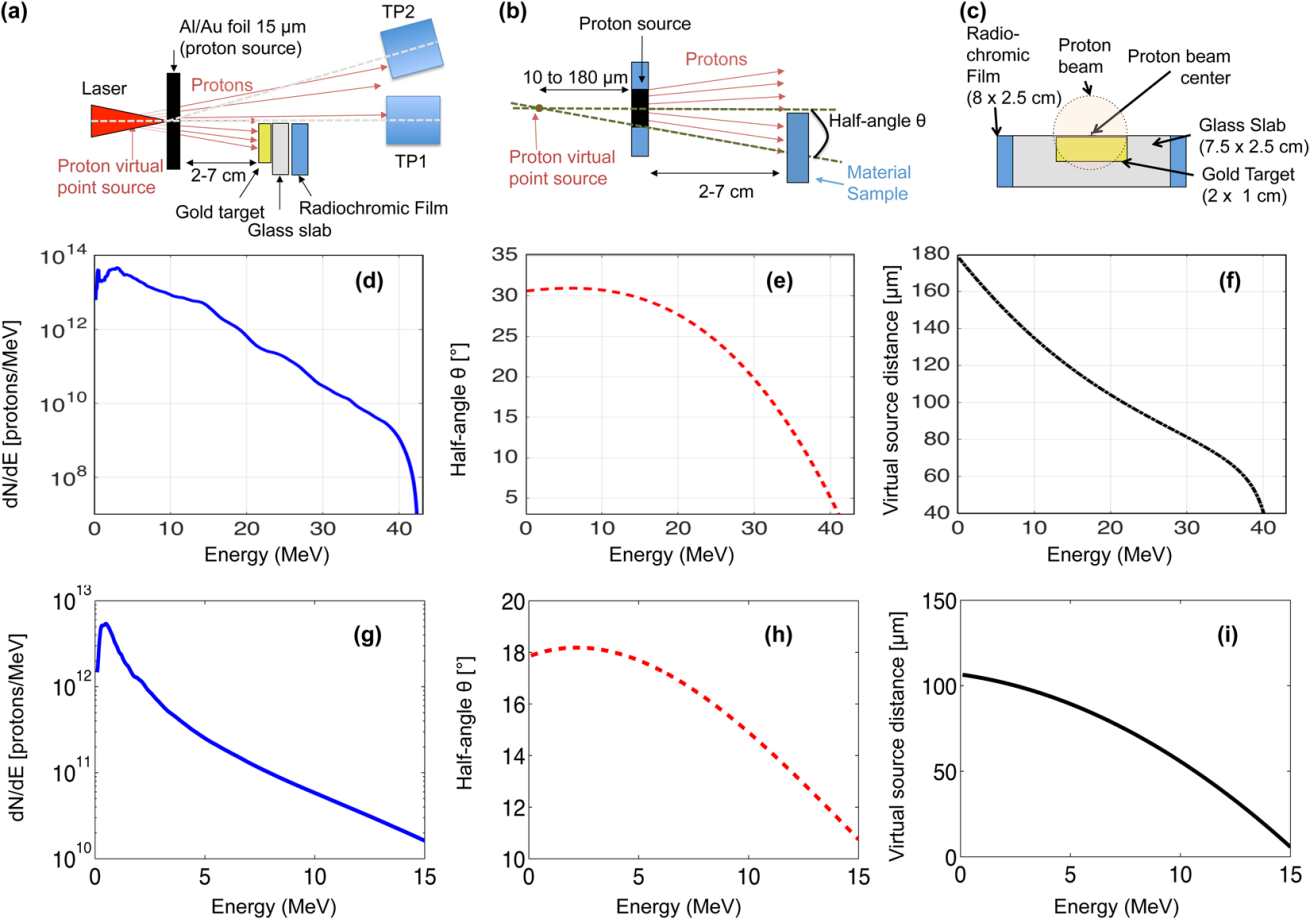
(**a**) Experimental set-up; (**b**) sketch of the modeling for the laser-driven proton source; (**c**) frontal view of the setup; (**d**) typical proton spectrum measured during the shots with the TP0° on the TITAN laser; (**e**) Half angle divergence (θ) vs proton beam energy measured on the TITAN laser; (**f**) Virtual source point distance against proton beam energy measured on the TITAN laser; (**g**) typical proton spectrum measured during the shots with the TP0° on the ELFIE laser; (**h**) Half angle divergence (θ) vs proton beam energy measured on the ELFIE laser; (**i**) Virtual source point distance against proton beam energy measured on the ELFIE laser.

The experiment was performed using the TITAN laser facility located at the Lawrence Livermore National Laboratory (LLNL), (Livermore, USA) and the ELFIE laser facility located at LULI (Palaiseau, France). The experimental set-up is shown in Fig. 8. The laser parameters were respectively for the TITAN laser an energy E~220 J, pulse duration τ = 700 fs, wavelength λ = 1.054 µm, and for the ELFIE laser an energy E~20 J, pulse duration τ = 350 fs, wavelength λ = 1.057 µm, both lasers were focused to about 8-10 µm focal spot diameter (FWHM), yielding an intensity I~5 10^19^–1 10^20^ W/cm on target. The Amplified Spontaneous Emission (ASE) has been measured to be <10^−6^ in contrast. As proton producing targets, i.e. the targets on which the laser was interacting, we used commercially available (manufactured by Goodfellow) solid 15 µm aluminum or gold targets in order to accelerate protons in the laser-forward direction using the TNSA^29^ mechanism. The aluminum target was used when producing gold nanoparticles, while the gold target was used when producing aluminum and copper nanoparticles. This was done in order to make sure that debris produced by the interaction would not interfere with the nanoparticle measurements.

The protons emitted from the foil (*proton source target*) were impinging into a second target (*working or plume target*), made of gold or aluminum or copper (gold 99.9% and 250 µm thickness manufactured by Goodfellow, copper 99.9% and 250 µm thickness manufactured by Goodfellow, aluminum 99.9% and 250 µm thickness manufactured by Goodfellow), which was placed on axis at a variable distance (see Fig. 8a). The second target made of gold (or copper or aluminum, depending on the nanoparticles to be obtained) was fixed on a glass substrate of dimensions 7.5 × 2.5 cm (see Fig. 8b). Behind the glass target we placed an RCF^72^ to make sure that the proton beam was correctly impinging the glass slab. A 5 micron aluminum foil (not shown in the figure) was positioned in between the source and the second target to protect it from undesired particles and radiation produced during the laser-matter interaction.

As diagnostics, we used two calibrated Thomson parabolas (TPs) and spectrometers located at 0° (TP1) and 9° (TP2) for the TITAN laser and 0° (TP1) and 16° (TP2) for the ELFIE laser, with respect to the main pulse laser axis to measure the forward generated proton spectrum. The TPs were placed respectively at a distance of 910 and 920 for the TITAN laser and 690 and 565 mm for the ELFIE laser from the proton source (distance to the entrance slit). Proton spectra measured by the TPs were readout in an absolute manner^73,74^ using Image Plates (BAS-TR 2025 from Fuji Photo Film Co. Ltd.) that were analyzed using a FUJIFILM FLA-7000 reader. Additional measurements of the proton spectra were obtained using Radio Chromic Films (RCFs) of the type HS that allowed obtaining a beam spatial distribution. On the shots the target was placed occupying only half of the proton beam so that the TP could readout the spectrum using the other half of the proton beam.

### Interaction simulations

The interaction between the laser-generated proton beam and the gold foil was modeled using a two-dimensional Energy Deposition Code that simulated the energy deposition phase and estimated the temperature reached by the proton heating. The code takes as input the experimental beam parameters presented in Fig. 8d–i (depending on the laser), which includes the number of protons (see a typical laser-generated proton spectrum in Fig. 8d for the TITAN laser and Fig. 8g for the ELFIE laser), the cone beam half-angle (see Fig. 8e for the TITAN laser and Fig. 8h for the ELFIE laser) and virtual source point position variation with energy (see Fig. 8f for the TITAN laser and Fig. 8i for the ELFIE laser). The laser-generated proton beam was modeled as the projection of a proton point source with diverging rays at a certain distance. The code considers a Gaussian transverse fluence profile and the energy deposition is calculated for our specific cone beam geometry using the stopping power tables available from the NIST-PSTAR^75^ database. We have inserted into the code the proton source as obtained in the same experimental conditions and as measured during the shots (the details are shown in Fig. 8d–i). All these beam characteristics (spectrum, half-angle and virtual point source position) were obtained experimentally using Thomson parabolas (TP) as proton spectrometers and Radiochromic films (RCF) for determining the half-angle and virtual source position for each proton energy. The divergence half angle of the proton rays (*θ*) has been adjusted depending on the considered proton energy as obtained in Ref. ^76^. Several simulations were run in order to find the most suitable distance in order to identify the optimum distance between proton source and the second gold target for catalyzing the above-described process using the TNSA spectra depicted in Fig. 8.

### Molecular Dynamics simulations

The LAMMPS (Large-scale Atomic/Molecular Massively Parallel Simulator) code was used to perform MD (Molecular Dynamics) simulations of the gold target systems. LAMMPS^77^ is a classical MD code that models an ensemble of particles in a liquid, solid, or gaseous state. It can model atomic, polymeric, biological, metallic, granular, and coarse-grained systems using a variety of force fields and boundary conditions.

The crystal structure of gold is the face centered cubic (FCC) with experimental lattice parameter a_Au_ = 4.078 Å. To set up the numerical model, initially, a crystalline FCC bulk system of N = 4000 gold atoms was considered (10 × 10 × 10 crystal unit cell). PBC (Periodic Boundary Conditions) were imposed to mimic an infinitely extended system in x, y and z directions. The LAMMPS code uses interatomic potentials to model interactions between atoms. In this case, the embedded-atom method (EAM)^78–80^ was used as the many-body potential scheme.

By performing an energy minimization of the system, we found the following values for the lattice parameter and the cohesion energy: a_Au_ = 4.080 Å, E_coes_ = −3.93 eV/atom. The simulation cell was cubic with: L_0_ = 40.80 Å. Then, the system was heated from the absolute zero temperature up to 500 °C in 200 ps by performing a MD simulation at hydrostatic constant pressure (P = 0 Pa). Subsequently a further MD simulation, taking both T and P as constant, was performed for a timeframe of 800 ps to have a relaxed bulk system with L_1_ = 41.22 Å and then a density ρ = 18.7 g/cm^3^ (reference ρ = 18.9 g/cm^3^).

The equations of motion used in the simulations are those of Shinoda *et al*. in ref. ^81^, which combine the hydrostatic equations of Martyna *et al*.^82^ with the strain energy proposed by Parrinello and Rahman in ref. ^83^. The time integration schemes closely follow the time-reversible measure-preserving Verlet and reversible reference system propagator algorithm integrators^84^.

For the setup we considered a bulk gold (100) surface; the surface was cleaved from the previous relaxed system corresponding to the (100) Miller plane. A vacuum space of 160 Å was inserted in the z direction to reduce the interaction between the free surfaces and to allow the cool down of the detached atoms and the eventual formation of clusters. The simulation cell was: L_x_ = L_y_ = 41.22 Å, L_z_ = 200 Å.

For the evaluation of the nanoparticle diameter versus temperature, the MD simulations were further extended for 300 ps for all the temperature ranges 2500 °C < T^*^ < 3500 °C. Below T* = 2500 °C no nanoparticles were detected. To estimate the nanoparticle diameter, we calculated the area of the surfaces delimited by the clusters (depicted as *C*_*1*_, *C*_*2*_, *C*_*3*_, *C*_*4*_ and *C*_*5*_ in the Fig. 7) using the algorithm implemented in the visualization software OVITO^85,86^; then we considered spheres of the same area and derived their diameters (see Fig. 11). For each temperature, the nanoparticle diameter was estimated and averaged in time.

### Morphological analysis

The morphological analysis on the nanostructured surfaces was conducted by SEM and AFM microscopies. AFM images were obtained using an ICON AFM microscope from Bruker working in tapping mode. Each image was taken with a resolution of 512 × 512 pixels and a frequency of about 1 Hz. For each sample, we scanned several areas in different windows such as 500 nm x 500 nm, 1 μm x 1 μm and 5 μm × 5 μm. Shape and dimensions of the NPs were analyzed conducting a statistical analysis on about 100 nanoparticles per image collected in typically 10 AFM images retrieved on the surface for the same shot. In most cases we repeated the shots in the same conditions and, after having assessed with the TPs that the proton dose was very similar to the previous shot, included those results in the statistics. The images were elaborated using the Nanoscope software (1.40 version from Bruker) to obtain a 3D structure and the particle volume using the Bearing analysis. The radius of each particle was evaluated assuming that the volume of a spherical particle is conserved during both, the deposition process and the interaction between a substrate and the AFM tip. The mean particle size and standard deviation was retrieved fitting the obtained Nanoscope data with a Gaussian distribution and performing equivalence calculations (see e.g. the OriginLab Software Manual^87^). Given the different quantities and bin sizes, the number of nanoparticles has been normalized in the histograms. SEM images were taken under a STEREOSCAN SEM microscope working with an energy of 20 keV.

Crystallinity characteristics of the surfaces were investigated by X-Ray Powder spectroscopy (XRD), using a monochromatic Bruker XRD spectrometer working with the copper kα line and using a 2Θ configuration at 3° of incident X-Ray beam to analyze the first 10 nanometers of the target surface. XRD spectra were analyzed with the EVA software for checking the crystallinity. A Gaussian model fit was used to evaluate the band centers and the full width at half maximum (FWHM) in order to obtain the crystallinity size.

### Extinction measurements and analysis

The UV-VIS extinction measurements were conducted on the particle deposited onto the microscope glasses. After the NPs deposition, the glasses were positioned between a light source and a spectrometer. A white lamp (Energetiq LDLS, Laser Driven Light Source) and an UV-VIS spectrometer (TRIAX 320 from Horiba-Jobyn–Yvon) for measuring the optical absorption measurements were placed normally to the glass. The white lamp was illuminating the entire region occupied by the NPs while, in transmission, the spectrometer was acquiring the spectra (the white lamp and spectrometer signal was transmitted over a series of optical lenses and fibers).

**Figure 9:**
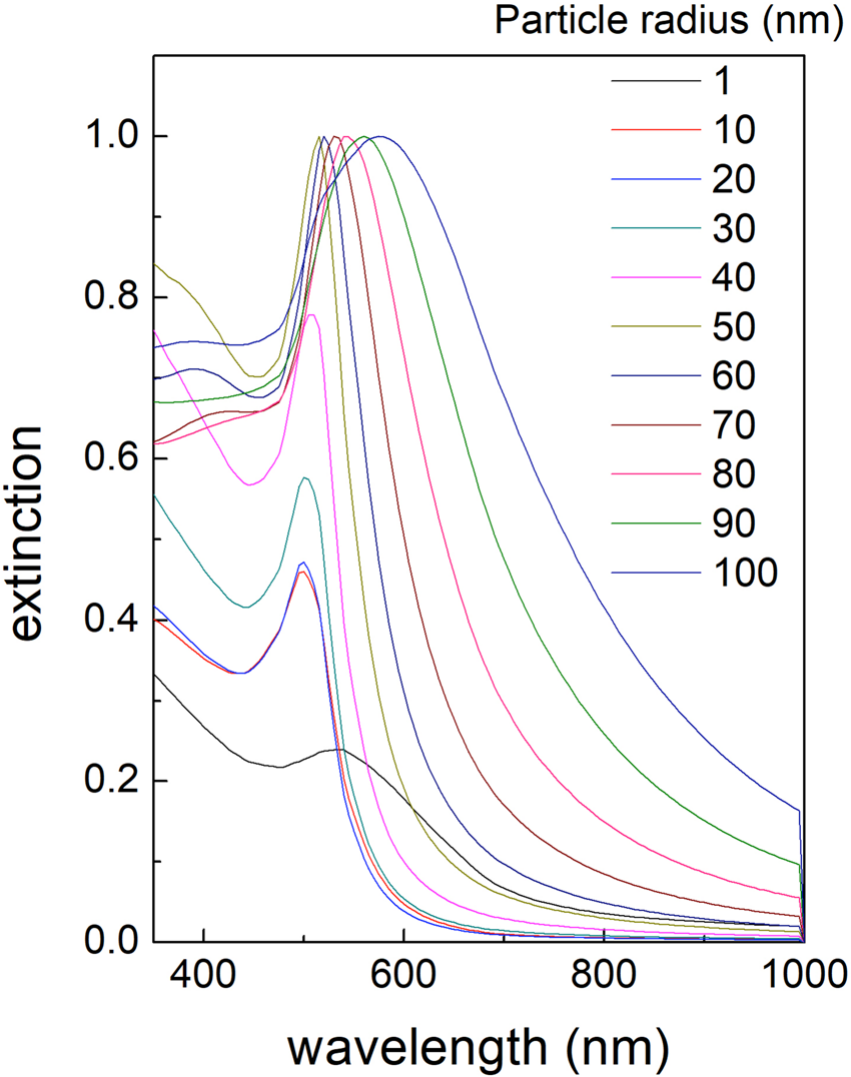
Extinction curves for gold nanoparticles deposited on glass using the Mie theory and Gans corrections.

Considering the transmitted spectra in all the UV-VIS range (300–800 nm), the extinction cross-section and optical absorption was evaluated using the standard equations:

1 $$\in (\lambda )=-\,Log\frac{{I}_{t}(\lambda )}{{I}_{0}(\lambda )}$$

2 $$a(\lambda )=1-\frac{{I}_{t}(\lambda )}{{I}_{0}(\lambda )}$$

where I_t_ and I_0_ are, respectively, the transmitted and source intensity at each wavelength. The obtained extinction spectra were assessed following the exact Mie Theory with Gans correction as indicated in^68,69^. All the software for the optical absorption analysis had been prepared and previously tested^71^. Theoretical spectra of gold nanoparticles as obtained using the theory are shown in Fig. 9.

**Figure 10:**
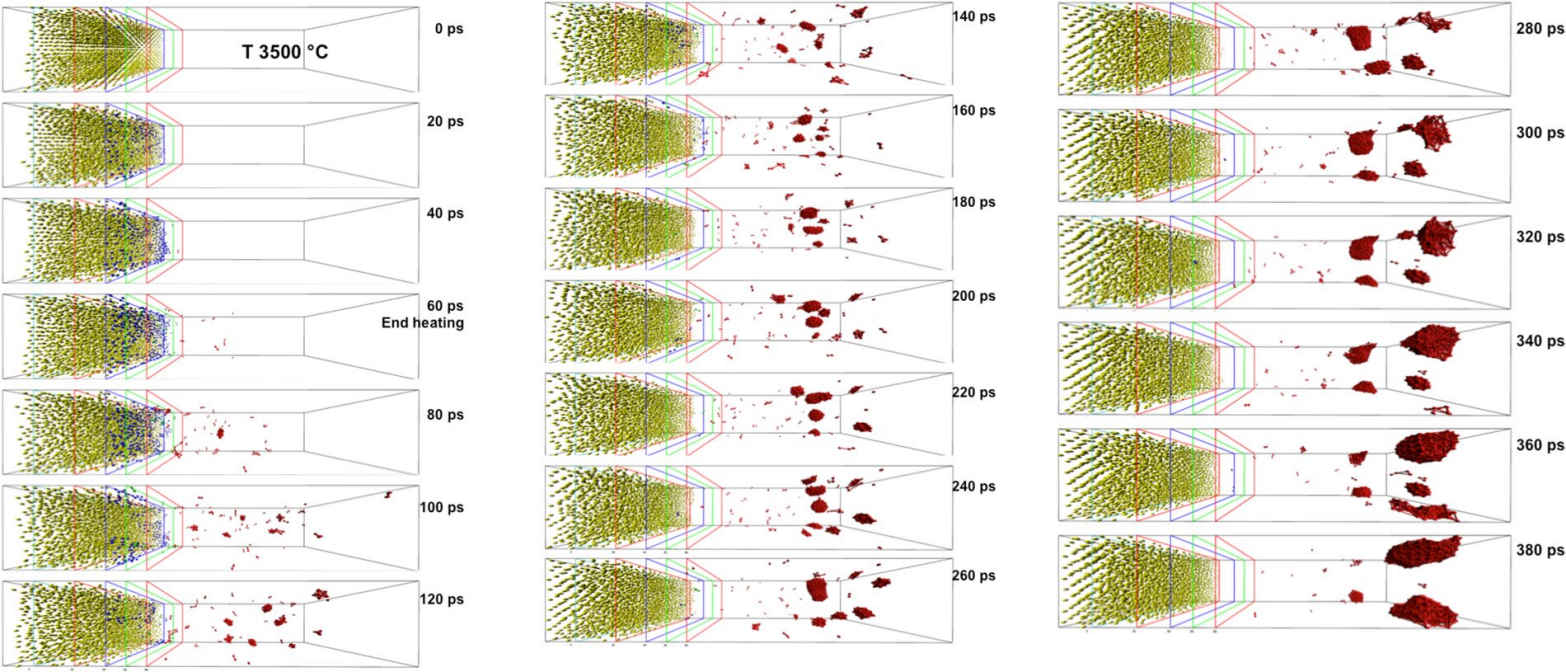
Longer Simulation runs showing the clustering process. Snapshots of a target heated at *T* = 3500 °C in 60 ps. A constant temperature is imposed for additional 320 ps. Images are produced at regular time intervals of 20 ps. At 80 ps some clusters are already formed; these clusters are used to compute the cluster diameter to compare with the experimental data. At 180 ps the clusters reach the cold zone and become aggregation points for even larger clusters. In the simulations with the lower temperature, the cold zone is reached at later times.

**Figure 11:**
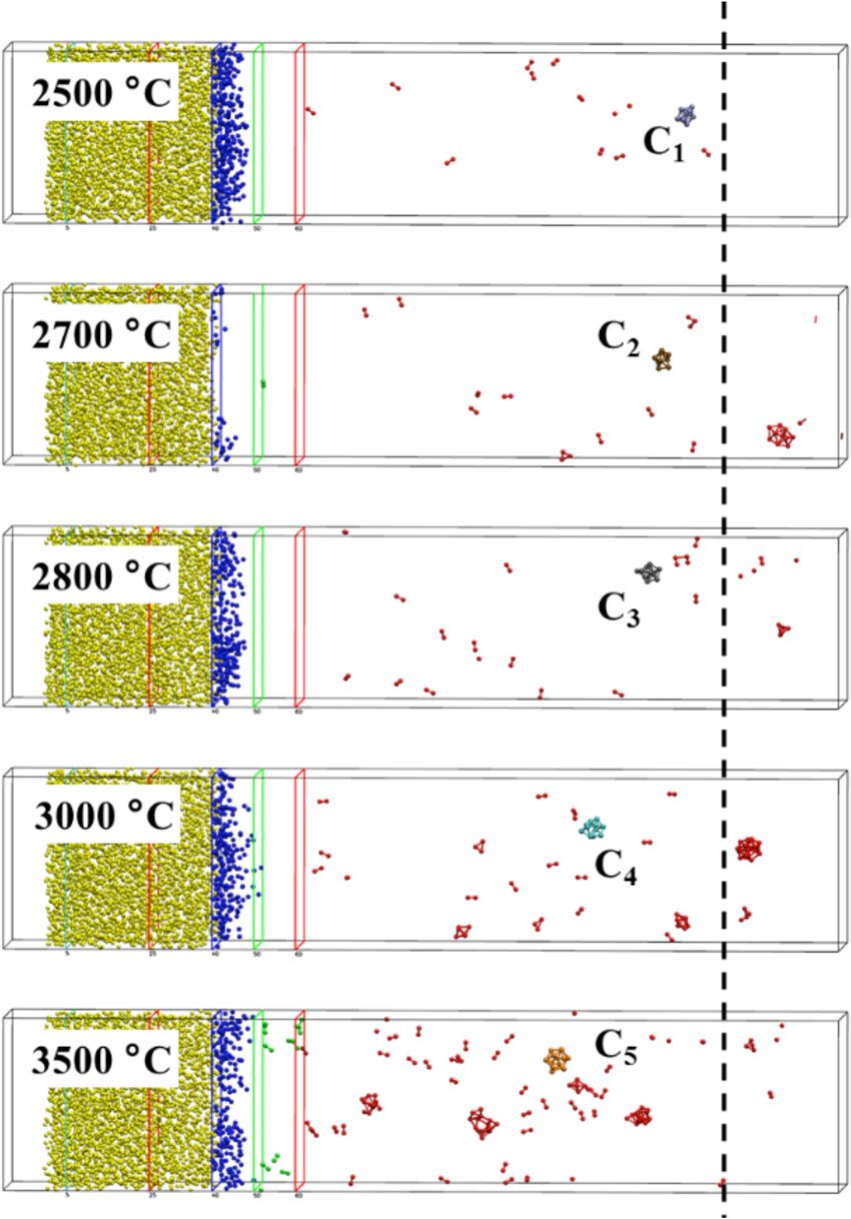
Longer Simulation runs showing the clustering process. Snapshots of the numerical system for MD simulations in the range 2500 °C < T^*^ < 3500 °C, in which the clusters are pointed out as C_1_, C_2_, C_3_, C_4_, and C_5_. The vertical black dashed line on the right indicates the point after which the clusters motion is slowed down.

**Figure 12:**
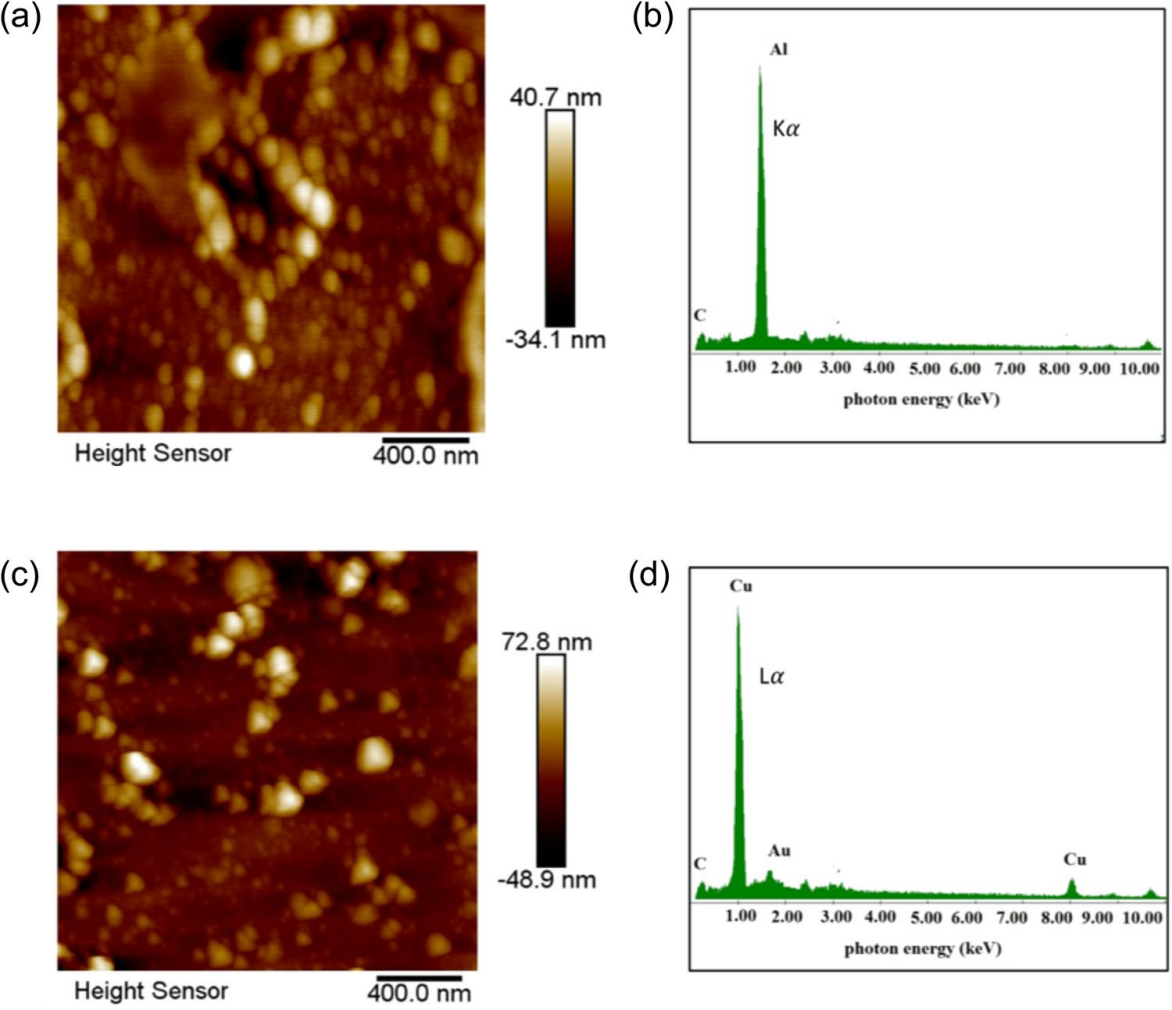
AFM and EDX analysis under SEM conditions for the aluminum and copper targets. (**a**) AFM image of a NP layer obtained using an aluminum proton-irradiated sample, at a distance of 2 cm from the particle source. The analyzed zone on the glass is at a 250 μm radial distance from the plume border. (**b**) EDX spectrum of the deposited aluminum layer, taken under SEM conditions. The peak at ∼ 1.5 keV indicates the K-alpha emission of aluminum. There are no significant signatures of other materials. (**c**) AFM image of a NP layer obtained using a copper proton-irradiated sample, at a distance of 2 cm from the particle source. The analyzed zone on the glass is at a 250 μm radial distance from the plume border. (**d**) EDX spectrum of the deposited copper layer, taken under SEM conditions. The peak at ∼ 0.9 keV indicates the L-alpha emission of Copper. There is a peak, significantly lower compared to the L-alpha peak of Copper, at ∼2.1 keV indicating the presence of gold in the deposited layer. This is likely due to the debris coming from the proton source that slightly contaminates the irradiated material sample.

### Video material

Different 3D animated video of the plume process as obtained by the LAMMPS code for different temperatures can be found here: http://www.afs.enea.it/giuseps/gold19/.

## Supplementary information

Supplementary Information.
